# C. J. Field *vs.* Ad. McGlumphy

**Published:** 1899-07

**Authors:** 


### C. J. FIELD *vs.* AD. McGLUMPHY.

Most of our readers will remember that we mentioned this case in our issue of March last (Vol. xix, p. 136).

On Monday, June 12th, the case came on to be heard on the application of the plaintiff for an alternative writ of mandamus.

The application was supported by affidavits of eminent physicians forming a volume of 153 printed pages.

The plaintiff's petition claims that the vaccination laws of the State inflict disease and sometimes death upon healthy children, and are therefore in violation of Art. xiv of the Constitution of the United States.

The testimony, which is exceedingly strong and apparently convincing, seems to establish the following points, viz.:

(1) That vaccination has no effect in protecting the vaccinee from smallpox.

(2) That the disease which the vaccinists claim is implanted by vaccination and to which Jenner gave the name "vaccinia" is ordinarily a more severe disease than ordinary small-pox.

(3) That severe vaccinia is a much more severe and dangerous disease than severe small-pox.

(4) That the dangers of small-pox have been greatly exaggerated and that properly treated it does not become infectious.

(5) That vaccination is often performed with variolous vaccine virus, and in such cases produces small-pox; of this many illustrations are cited, and (pp. 30, 31) it is cogently argued that ten of the cases of small-pox, in two of which death resulted, reported by Dr. D. Welch, physician to the Municipal Hospital of Philadelphia, in his report for the year 1895, were cases in which Dr. Welch had himself implanted the small-pox with his vaccination.

A rule *nisi* was granted and the need for enlightening the public upon the subject is illustrated by the fact that in the course of the argument one of the Judges asked Mr. Beasley (of counsel for the plaintiff) whether the refusal of admission to the school was not like refusing admission to a child with measles. It was on Mr. Beasley's pointing out that the two cases were totally unlike, as this was the refusal of admission to a healthy child, that the rule *nisi* was granted.

Now comes the tug of war and the main expense; and we earnestly renew the invitation given in our issue of last March to all persons who can afford to do so to contribute to the expense of the case which, if it goes to the Supreme Court of the United States and be decided, as we think it must be, adversely to compulsory vaccination, will dispose of all laws to that effect in all the States.

The volume of testimony in this case is a repertory of the subject, and all persons who subscribe $2.00 or upwards to the expenses of the suit will receive a copy of this testimony on signifying their desire to do so on sending their subscriptions. Remittances may be made to C. Oscar Beasley, attorney, No. 311 Fidelity Building, 112 North Broad street, Philadelphia, Pa.; R. N. Taylor, Esq., 35 South Front street, Philadelphia, Pa., or to Dr. M. R. Leverson, Fort Hamilton, N. Y.

